# A critical view on transgenerational epigenetic inheritance in humans

**DOI:** 10.1038/s41467-018-05445-5

**Published:** 2018-07-30

**Authors:** Bernhard Horsthemke

**Affiliations:** Institut für Humangenetik, Universitätsklinikum Essen and Universität Duisburg-Essen, 45147 Essen, Germany

## Abstract

Transgenerational epigenetic inheritance refers to the transmission of epigenetic information through the germline. While it has been observed in plants, nematodes and fruit flies, its occurrence in mammals—and humans in particular—is the matter of controversial debate, mostly because the study of transgenerational epigenetic inheritance is confounded by genetic, ecological and cultural inheritance. In this comment, I discuss the phenomenon of transgenerational epigenetic inheritance and the difficulty of providing conclusive proof for it in experimental and observational studies.

## Epigenetics and cellular inheritance

Even though all somatic cells of a multicellular organism have the same genome, different cell types have different transcriptomes (set of all expressed RNA molecules), different proteomes (set of all proteins) and, hence, different functions. Cell differentiation during embryonic development requires the activation and repression of specific sets of genes by the action of cell lineage defining transcription factors. Within a cell lineage, gene activity states are often maintained over several rounds of cell divisions (a phenomenon called “cellular memory” or “cellular inheritance”). Since the rediscovery of epigenetics some 30 years ago (it was originally proposed by Conrad Hal Waddington^1^ in the early 1940s), cellular inheritance has been attributed to gene regulatory feedback loops, chromatin modifications (DNA methylation and histone modifications) as well as long-lived non-coding RNA molecules, which collectively are called the “epigenome”. Among the different chromatin modifications, DNA methylation and polycomb-mediated silencing are probably the most stable ones and endow genomes with the ability to impose silencing of transcription of specific sequences even in the presence of all of the factors required for their expression^2^.

## Defining transgenerational epigenetic inheritance

The metastability of the epigenome explains why development is both plastic and canalized, as originally proposed by Waddington. Although epigenetics deals only with the cellular inheritance of chromatin and gene expression states, it has been proposed that epigenetic features could also be transmitted through the germline and persist in subsequent generations. The widespread interest in “transgenerational epigenetic inheritance” is nourished by the hope that epigenetic mechanisms might provide a basis for the inheritance of acquired traits. Yes, Lamarck has never been dead and every so often raises his head, this time with the help of epigenetics.

Although acquired traits concerning body or brain functions can be written down in the epigenome of a cell, they cannot easily be transmitted from one generation to the next. For this to occur, these epigenetic changes would have to manifest in the germ cells as well, which in mammals are separated from somatic cells by the so-called Weismann barrier. Further, the chromatin is extensively reshaped during germ cell differentiation as well as during the development of totipotent cells after fertilization, even though some loci appear to escape epigenetic reprogramming in the germline^3^. Long-lived RNA molecules appear to be less affected by these barriers and therefore more likely to carry epigenetic information across generations^4^, although the mechanisms are largely unsolved.

## Evidence for transgenerational epigenetic inheritance

In the past 10 years, numerous reports on transgenerational responses to environmental or metabolic factors in mice and rats have been published (for a comprehensive review, see refs. ^4–7^). The factors include endocrine disruptors, high fat diet, obesity, diabetes, undernourishment as well as trauma. These studies investigated DNA methylation, sperm RNA or both. Wei et al.^8^, for example, found that streptozotocin-induced prediabetes in male mice affected DNA methylation patterns in sperm as well as pancreatic islets of F1 and F2 offspring. Gapp et al.^9^ found that traumatic stress in early life altered behavioral and metabolic processes in the progeny and that injection of sperm RNAs from traumatized males into fertilized wild-type oocytes reproduced the alterations in the resulting offspring. While most of published studies are technically sound, the majority still await independent confirmation; studies on transgenerational effects of endocrine disruptors and of high fat diet on the DNA methylome have recently been challenged by others^10,11^.

In humans, epidemiological studies have linked food supply in the grandparental generation to health outcomes in the grandchildren^12^. An indirect study based on DNA methylation and polymorphism analyses has suggested that sporadic imprinting defects in Prader–Willi syndrome are due to the inheritance of a grandmaternal methylation imprint through the male germline^13^. Because of the uniqueness of these human cohorts these findings still await independent replication. Most cases of segregation of abnormal DNA methylation patterns in families with rare diseases, however, turned out to be caused by an underlying genetic variant^14–16^ (see below).

## Fetal programming and intergenerational inheritance

**Fig. 1:**
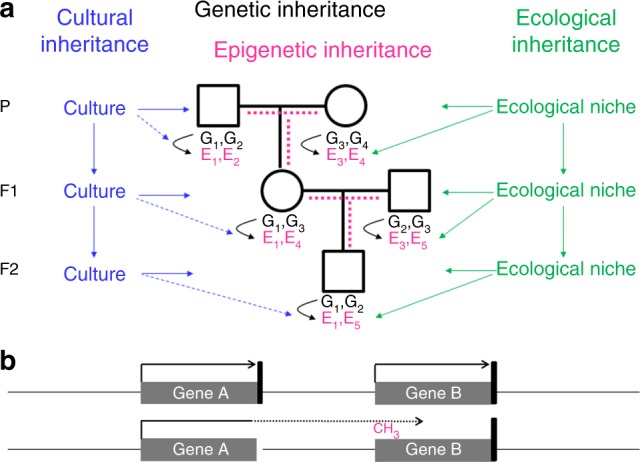
Transgenerational inheritance systems. **a** Offspring inherit from their parents genes (black), the environment (green) and culture (blue). Genes and the environment affect the epigenome (magenta) and the phenotype^22^. Culture also affects the phenotype, but at present there is no evidence for a direct effect of culture on the epigenome (broken blue lines). It is a matter of debate, how much epigenetic information is inherited through the germline (broken magenta lines). G genetic variant, E epigenetic variant. **b** An epimutation (promoter methylation and silencing of gene B in this example) often results from aberrant read-through transcription from a mutant neighboring gene, either in sense orientation as shown here or in antisense orientation. The presence of such a secondary epimutation in several generations of a family mimics transgenerational epigenetic inheritance, although it in fact represents genetic inheritance. Black arrow, transcription; black vertical bar, transcription termination signal; broken arrow, read-through transcription

Genetic inheritance alone cannot fully explain why we resemble our parents. In addition to genes, we inherited from our parents the environment and culture, which in parts have been constructed by the previous generations (Fig. 1a). A specific form of the environment is our mother’s womb, to which we were exposed during the first 9 months of our life. The maternal environment can have long-lasting effects on our health. In the Dutch hunger winter, for example, severe undernourishment affected pregnant women, their unborn offspring and the offspring’s fetal germ cells. The increased incidence of cardiovascular and metabolic disease observed in F1 adults^17^ is not due to the transmission of epigenetic information through the maternal germline, but a direct consequence of the exposure in utero, a phenomenon called “fetal programming” or—if fetal germ cells and F2 offspring are affected—“intergenerational inheritance”.

## Secondary epimutations

An intrinsic feature of the epigenome is that it is affected by genes and the environment. The effect of culture is currently less clear; if it does have an effect, then probably indirectly by niche construction. Parents and offspring may share the same epigenomic features, but it is extremely difficult to divide out whether these features have been transmitted through the germline or established anew in each generation by the action of shared genes and shared environments.

Several studies^14–16^ including a recent study by Guéant et al.^18^ in this journal, have reported the co-segregation of an abnormal DNA methylation pattern (called “epimutation”) with a rare disease in two or more generations of certain families. In these cases, the abnormal DNA methylation of the gene under investigation was linked to a mutation in a neighboring gene that removed the transcription termination signal (Fig. 1b). As a consequence, transcription from this gene extended into the gene under investigation, causing abnormal promoter methylation and gene silencing. In contrast to a primary epimutation, which occurs independently of any DNA sequence change, this is a secondary epimutation^19^, which strictly depends on the expression of the mutated neighboring gene. If this gene is expressed also in the germline, as in the families described by Guéant et al.^18^, the epimutation is also found in germ cells , which should not be mistaken for transgenerational epigenetic inheritance. True transgenerational epigenetic inheritance would depend on the presence of a primary epimutation in the germ cells having a direct effect on the offspring’s phenotype.

## Roadmap to proving transgenerational epigenetic inheritance

1. Rule out genetic, ecological and cultural inheritance. For studies in mice and rats, inbred strains and strictly controlled environments need to be used. When a pregnant female animal is exposed to a specific environmental stimulus, F3 offspring and subsequent generations must be studied in order to exclude a direct effect of the stimulus on the embryos’ somatic cells and germ cells. Even more desirable is the use of in vitro fertilization (IVF), embryo transfer and foster mothers. When a male animal is exposed to an environmental stimulus, F2 offspring must be studied in order to exclude transient effects on germ cells. To ensure that any phenotype is exclusively transmitted via gametes, IVF must be used, controlling for possible artifacts relating to IVF. In contrast with laboratory animals, it is impossible to rule out ecological and cultural inheritance in humans, but genetic effects should and can be excluded. If an epimutation apparently follows Mendelian inheritance patterns, be cautious: you are more likely looking at a secondary epimutation and genetic inheritance. Study the haplotype background of the epimutation: if in a given family it is always on the same haplotype, you are again most likely dealing with a secondary epimutation. Do whole genome sequencing, as Guéant et al.^18^ did, to search for a genetic variant that might have caused the epimutation and be aware that this variant might be distantly located. Good spots to start looking are the two neighboring genes, where a mutation might cause transcriptional read-through in sense or antisense orientation into the locus under investigation. Unfortunately, if you don’t find anything, you still cannot be 100% sure that a genetic variant does not exist.
2. Identify the responsible epigenetic factor in the germ cells. Admittedly, this is easier said than done, especially in female germ cells, which are scarse or unavailable. Be aware that germ cell preparations may be contaminated with somatic cells or somatic DNA. Use swim-up (sperm) or micromanipulation techniques to purify germ cells to the highest purity. Exclude the presence of somatic cells and somatic DNA by molecular testing, for example by methylation analysis of imprinted genes, which are fully methylated or fully unmethylated only in germ cells.
3. Demonstrate that the epigenetic factor in the germ cells is responsible for the phenotypic effect in the next generation. If possible, remove the factor from the affected germ cells and demonstrate that the effect is lost. Add the factor to control germ cells and demonstrate that the effect is gained. While RNA molecules can and have been extracted from sperm of exposed animals and injected into control zygotes^9^, DNA methylation and histone modifications cannot easily be manipulated (although CRISPR/Cas9-based epigenome editors are being developed and used for this purpose^20^), and all of these experiments can hardly be done in humans. In light of these problems, this might currently be too much to ask for to prove transgenerational epigenetic inheritance in humans, but should, nevertheless, be kept in mind and discussed.

## Transgenerational inheritance in the light of evolution

In plants, nematodes and fruit flies, transgenerational epigenetic inheritance is well documented. It has been argued that this form of inheritance may permit a population to adapt to fluctuating environments. The question is whether this is also true for mammals and, particularly, humans. Almost all of the experimental mouse models and the few observations in humans concern deleterious traits (congenital malformations, anxiety, glucose intolerance, obesity, cardiovascular diseases, cancer and premature death); an exception appears to be hepatic wound healing^21^. This may, at least in part, be due to reporting bias, as negative effects are easier to spot than positive effects, but overall casts doubt on an adaptive role of transgenerational epigenetic inheritance in these cases. That transgenerational inheritance of chromatin marks is so rarely observed in mammals may be a side effect of the extensive epigenetic reprogramming required for germ cell development and early embryogenesis in mammals, which could also serve as a mechanism to prevent the transmission of environmental insults that animals have encountered during their life.

More generally speaking, the transmission of epigenetic information between generations reduces developmental plasticity and canalizes the development of offspring into a particular direction. This may help fast-reproducing animals to rapidly adapt to a new environment and increase population size. If, however, the “anticipated” environment does not match the actual environment, offspring will be maladapted and have reduced reproductive fitness. This is especially true for humans, who are likely to encounter different environments in their long life.

In conclusion, in my opinion, even if the molecular mechanisms exist to transmit epigenetic information across generations in humans, it is very likely that the transgenerational transmission of culture by communication, imitation, teaching and learning surpasses the effects of epigenetic inheritance and our ability to detect this phenomenon. Cultural inheritance has certainly had an adaptive role in the evolution of our species, but the evidence for transgenerational epigenetic inheritance, as laid out above, is not (yet) conclusive. For now, I remain skeptical.
